# Specific Management of Post-Chikungunya Rheumatic Disorders: A Retrospective Study of 159 Cases in Reunion Island from 2006-2012

**DOI:** 10.1371/journal.pntd.0003603

**Published:** 2015-03-11

**Authors:** Emilie Javelle, Anne Ribera, Isabelle Degasne, Bernard-Alex Gaüzère, Catherine Marimoutou, Fabrice Simon

**Affiliations:** 1 Department of Tropical and Infectious Diseases, Laveran Military Teaching Hospital, Marseille, France; 2 Private Rheumatology Office, Saint Denis, La Réunion, France; 3 Department of Rheumatology, Centre Hospitalier Universitaire de La Réunion, Hôpital Felix Guyon, Saint Denis, La Réunion, France; 4 Intensive Care Unit, Centre Hospitalier Universitaire de La Réunion, Hôpital Felix Guyon, Saint Denis, La Réunion, France; 5 French Army Centre for Epidemiology and Public Health (“IRBA”), Marseille, France; University of Minnesota, UNITED STATES

## Abstract

**Background:**

Since 2003, the tropical arthritogenic chikungunya (CHIK) virus has become an increasingly medical and economic burden in affected areas as it can often result in long-term disabilities. The clinical spectrum of post-CHIK (pCHIK) rheumatic disorders is wide. Evidence-based recommendations are needed to help physicians manage the treatment of afflicted patients.

**Patients and methods:**

We conducted a 6-year case series retrospective study in Reunion Island of patients referred to a rheumatologist due to continuous rheumatic or musculoskeletal pains that persisted following CHIK infection. These various disorders were documented in terms of their clinical and therapeutic courses. Post-CHIK de novo chronic inflammatory rheumatisms (CIRs) were identified according to validated criteria.

**Results:**

We reviewed 159 patient medical files. Ninety-four patients (59%) who were free of any articular disorder prior to CHIK met the CIR criteria: rheumatoid arthritis (n=40), spondyloarthritis (n=33), undifferentiated polyarthritis (n=21). Bone lesions detectable by radiography occurred in half of the patients (median time: 3.5 years pCHIK). A positive therapeutic response was achieved in 54 out of the 72 patients (75%) who were treated with methotrexate (MTX). Twelve out of the 92 patients (13%) received immunomodulatory biologic agents due to failure of contra-indication of MTX treatment. Other patients mainly presented with mechanical shoulder or knee disorders, bilateral distal polyarthralgia that was frequently associated with oedema at the extremities and tunnel syndromes. These pCHIK musculoskeletal disorders (MSDs) were managed with pain-killers, local and/or general anti-inflammatory drugs, and physiotherapy.

**Conclusion:**

Rheumatologists in Reunion Island managed CHIK rheumatic disorders in a pragmatic manner following the outbreak in 2006. This retrospective study describes the common mechanical and inflammatory pCHIK disorders. We provide a diagnostic and therapeutic algorithm to help physicians deal with chronic patients, and to limit both functional and economic impacts. The therapeutic indication of MTX in pCHIK CIR could be approved in future efficacy trials.

## Author Summary

With a 6-year insight, we extensively and retrospectively describe clinical profiles and specific treatments of mechanical and inflammatory post-chikungunya rheumatic disorders. In the current context of chikungunya’s global spread, we provide the first diagnostic and therapeutic algorithm to guide physicians according to the amount of time that has elapsed since the acute CHIK infection.

## Introduction

Fifty years after its first tropical description in the Newala district of Tanganyika [1,2], chikungunya (CHIK) has re-emerged extensively, resulting in 1.4 to 6.5 million infected individuals between 2004 and 2014 in Africa, as well as regions within the Indian Ocean, Southeast Asia, the Pacific Islands and Europe [3]. Its first autochthonous transmission in the intertropical Americas was identified at the end of 2013 [4], and six months later this turned into a large outbreak in most of the Caribbean Islands that has now reached northern South America [5] and the United States [6]. Currently, this arboviral disease represents a pressing threat to public health in large areas of the American and European continents that are colonized by the primary disease vector: the *Aedes* mosquito [4,7].

CHIK is usually characterized by an acute febrile and sometimes eruptive polyarthritis, commonly followed by persistent rheumatologic and general disabling symptoms [8]. Historically, post-CHIK (pCHIK) rheumatic disorders were first described in South Africa after a local outbreak at the end of the 1970s. In 1979, Fourie and Morrison first reported a pCHIK rheumatoid arthritic syndrome [9], and in 1983 Brighton *et al*. highlighted a high prevalence of chronic polyarthralgia or stiffness occurring three years after disease onset [10], with one case of destructive pCHIK polyarthritis [11]. After minimal exposure in the literature over the last 20 years, the wide clinical spectrum of pCHIK rheumatic disorders has been rediscovered [12–22]. During its re-emergence in the past 10 years, 92 published articles related to chronic pCHIK status have been indexed in PubMed [keywords: “chikungunya” and “chronic”, last search on October 24^th^, 2014], but there are no available evidence-based guidelines with standardized definitions and treatment recommendations. While the overall proportion of patients with chronic symptoms diminishes over time after CHIK onset (from 100 to 88% during the first 6 weeks, to less than 50% after 3–5 years, with variable results depending on the study), the time required to return to pre-CHIK status is still uncertain, as some infected individuals remain symptomatic for 6 to 8 years post-infection [23,24].

Simple analgesics and/or non-steroidal anti-inflammatory drugs (NSAIDs) provide relief in most patients [25], but better-targeted drugs are clearly needed to treat inflammatory rheumatic disorders. Hydroxychloroquine and ribavirin were not effective [26–28], but methotrexate (MTX) was of benefit in pCHIK inflammatory polyarthritis [29–31]. MTX efficacy is supported by scientific data that indicates active monocyte/macrophage trafficking into the synovial tissue of chronic patients, possibly maintained by a local viral persistence [32].

Thus, the current challenge for physicians in epidemic areas is to identify and diagnose pCHIK rheumatic disorders and to provide the optimal treatment in order to prevent perpetuation or progression to a potentially destructive disease course.

To answer this real-world need, we retrospectively documented the clinical and therapeutic courses of rheumatic and musculoskeletal pCHIK disorders (pCHIK-RMSD) that were referred to rheumatologists in Reunion Island over a 6-year period following the 2005–2006 epidemic. We have summarized this practical experience in an algorithm for disease management purposes.

## Methods

### Ethics statement

Data were anonymously analyzed and reported. The study was approved by the research ethical committee of Laveran Military Teaching hospital (registration n° 2014-PRSimon).

### Design

This retrospective study was carried out at two referring rheumatologic practices: University Hospital (“CHU”) Félix Guyon and the private office of a rheumatologist in Saint-Denis, the capital of Reunion Island, which was affected by a massive CHIK outbreak in 2005–2006.

### Population

All eligible participants were older than 16 years of age and had been referred to the specialist for joint pain that persisted for more than 4 months following typical clinical acute CHIK infection [33], which was assumed to be related to CHIK-RMSD. Biological confirmation of CHIK infection was obtained for all patients, either by detection of CHIK virus RNA in the blood during the acute stage by reverse-transcriptase polymerase chain reaction (RT-PCR), or the presence of anti-CHIK virus-specific immunoglobulin M and/or G (depending on the delay relative to the acute CHIK infection) as detected by enzyme-linked immunosorbent assay (ELISA) using CHIK virus antigens produced by the French Referent National Centre for Arbovirus, Marseille, IRBA, France.

### Data collection

Data were collected anonymously and retrospectively from January to May of 2012, based on patient medical files using a structured questionnaire developed for the purposes of this study. Patient details were recorded, including factors considered to be predictors of non-recovery after CHIK according to the literature: age (>45 year), female gender, previous history of an osteoarthritic event, and severe acute CHIK [12,13,18,22,29,34]. Details on CHIK-RMSD included the date of the first visit to the rheumatologist, the clinical and biological histories, imaging features, and treatments since the acute CHIK infection. We recorded the following clinical data: i) joint involvement: small joints (metacarpophalangeal, proximal and distal interphalangeal, metatarsophalangeal, thumb interphalangeal joints and wrists), large joints (shoulders, elbows, hips, knees and ankles), vertebral, sacroiliac, temporomandibular and sternocostoclavicular areas; ii) joint count: polyarticular if more than 4 (oligoarticular if 4 or less); iii) articular inflammatory signs or symptoms defining arthritis: synovitis, warmth and/or redness over the joint (“hot” joint), prolonged morning stiffness (> 30 minutes), inflammatory pain (which improved with exercise, or worsened after rest or during the night), or arthritis that was clinically distinguished from oedema without join effusion; iv) periarticular involvement: enthesitis (inflammation of the tendon or ligament insertion into bone: Achilles tendonitis, plantar fasciitis), tenosynovitis, periostitis (tibial and ischial tuberosities inflammation), tendinopathy, bursitis, myalgia and neural tunnel syndrome. Hyperuricemia and vitamin D deficiency were determined using standard biological assays, as were the elevated erythrocyte sedimentation rate (ESR), and the levels of C-reactive protein (CRP), antinuclear antibodies (ANA), rheumatoid factor (RF), anti-citrullinated peptide autoantibodies 2 (ACPA2) and HLA B27 positive status, when required for scoring. The treatments were classified as follows: painkillers; oral or topical non-steroidal anti-inflammatory drugs (NSAIDs); oral or intra-articular corticosteroids; conventional disease-modifying anti-rheumatic drugs (DMARDs) including MTX (7.5–25 mg/week), hydroxychloroquine (200 mg/day), leflunomide (10–20 mg/day), sulfasalazine (1.5–3 g/day); immune-modulating biologic agents including anti-TNF, a B-lymphocyte depletion agent (rituximab), and an interleukin-6 receptor inhibitor (tocilizumab) for indications and at dosages recommended by national guidlines (former and revised text) [35,36]; physiotherapy; orthopaedic braces; complementary therapeutics such as vitamin D supplementation or gout treatment using colchicine with a urate-lowering diet, such as a xanthine oxidase inhibitor (allopurinol) or uricosurics.

### Data analysis

Patients without previously defined arthritis were classified as pCHIK musculoskeletal disorders (pCHIK-MSD) that were either loco-regional or diffuse (if > 4 painful areas). For patients with arthritis, we separated crystalline and non-crystalline rheumatic disorders. Crystalline arthritis was defined as a convincing clinical presentation associated with hyperuricemia that improved after gout treatment. Patients presenting non-crystalline polyarthritis were classified as chronic inflammatory rheumatism (CIR). CIRs were categorized into 3 groups (Table 1) [37,38]. Patients who had not previously displayed rheumatic symptoms and developed CIR immediately after CHIK were classified as *de novo* pCHIK-CIR.

**Table 1:** Definitions of chronic inflammatory rheumatisms (CIR): rheumatoid arthritis according to the 2010 American College of Rheumatology/European League Against Rheumatism (ACR/EULAR) criteria [37]; spondyloarthritis according the European Spondyloarthropathy Study Group (ESSG) Classification [38]; undifferentiated polyarthritis (own study criteria).

	Chronic inflammatory rhumatisms	
Rheumatoid arthritis (RA)	Spondyloarthritis (SA)	Undifferentiated polyarthritis (UP)
Unexplained synovitis in at least 1 joint + score ≥ 6/10	At least 1 major + 1 minor criteria	> 4 joints arthritis + duration of symptoms ≥ 6 weeks + absence of alternative diagnosis
**A. Joint involvement** *	**A. Major criteria**	**A. Arthritis** = 1 inflammatory criteria:
1 large joint = 0	1) Inflammatory back pain if 4/5 criteria:	- Synovitis
2–10 large joints = 1	- Onset of back discomfort before the age of 40 years	- Warmth and/or redness over joint (“hot” joint)
1–3 small joints (with or without involvement of large joints) = 2	- Insidious onset	- Prolonged morning stiffness > 30 minutes
4–10 small joints (with or without involvement of large joints) = 3	- Persistence for at least 3 months	- Inflammatory pain: improved with exercise or worse after rest or during the night
>10 joints (at least 1 small joint)** = 5	- Associated with morning stiffness	
**B. Serology (at least 1 test result is needed)** ***	- Improvement with exercise	
Negative RF *and* negative ACPA = 0	2) Synovitis either asymmetric or predominant in the lower limbs:	**B. Criteria of RA and SA not fulfilled and elimination of common causes of polyarthritis:** gout, auto-immune disorders, dysthyroidism, chronic viral hepatitis, sarcoidosis, etc.
Low-positive RF *or* low-positive ACPA = 2	- warmth over a joint or effusion	
High-positive RF *or* high-positive ACPA = 3	- Dactylitis (inflammation of an entire digit “sausage digit”)	
**C. Acute-phase reactants (at least 1 test result is needed)** ****	**B. Minor criteria**	NB: joint effusion with inflammatory criteria defines effusive arthritis and differs from swollen joint without arthritis.
Normal CRP and normal ESR = 0	- Psoriasis	
Abnormal CRP or abnormal ESR = 1	- Inflammatory bowel disease	
**D. Duration of symptoms (self-reported)**	- Urethritis, cervicitis, or acute diarrhea within one month before arthritis	
<6 weeks = 0	- Alternating buttock pain	
≥6 weeks = 1	- Enthesitis	
	- Sacroiliitis as determined on imaging of the pelvic region	
	- Positive family history	

* Joint involvement refers to any swollen or tender joint on examination, which may be confirmed by imaging evidence of synovitis. DIP, first CMC, and first MTP are excluded from assessment. Categories of joint distribution are classified according to the location and number of involved joints, with placement into the highest category possible based on the pattern of joint involvement. “Large joints” refers to shoulders, elbows, hips, knees, and ankles. “Small joints” refers to the MC, PIP, second through fifth MTP, thumb IP joints, and wrists.

** In this category, at least 1 of the involved joints must be a small joint; the other joints can include any combination of large and additional small joints, as well as other joints not specifically listed (e.g., temporomandibular, acromioclavicular, sternoclavicular…).

*** Negative refers to international unit (IU) values that are less than or equal to the upper limit of normal (ULN) for the laboratory and assay; low-positive refers to IU values that are higher than the ULN but ≤ 3 times the ULN for the laboratory and assay; high-positive refers to IU values that are > 3 times the ULN for the laboratory and assay. Where RF information is only available as positive or negative, a positive result should be scored as low-positive for RF.

****Normal/abnormal is determined by local laboratory standards.

IP = interphalageal;

PIP = proximal interphalangeal;

DIP = distal interphalangeal;

CMC = carpometacarpal;

MCP = metacarpophalangeal;

MTP = metatarsophalangeal;

CRP = C-reactive protein;

ESR = erythrocyte sedimentation rate;

ACPA = anti-citrullinated protein antibody.

We measured the median period from acute CHIK to the first rheumatology consultation for the pCHIK-CIR and pCHIK-MSD patient groups. The severity of all CHIK-RMSDs was evaluated based on the level of joint destruction or deformation (chondrolysis, joint space reduction, juxta-articular osteopenia, subluxation, bony erosion or destruction, sacroiliitis) detected in the imaging tests, and based on the need for an internal or external orthopaedic brace. The functional burden was estimated based on a history of being unable to work or requiring adjustment, self-estimated significant reduction of daily activities (as a percentage of reduction) [13] and initiation of psychological follow-up or antidepressant medical treatment since 2006 due to CHIK.

For each patient, we recorded the therapeutic strategy, including corticotherapy, and the outcome after complete drug withdrawal (the type of response at 3 and 6 months following therapeutic interruption). Rheumatologists treated pCHIK-CIR patients with MTX at the recommended dosage and routes of administration based upon the 2008 ACR Recommendations for Rheumatoid Arthritis Treatments [39] and French national guidelines [35,36]. We defined a positive response to MTX if there was no further need for a drug switch or escalation (treatment in association with other DMARDs). Recovery after MTX was defined as the absence of relapse more than 6 months after MTX withdrawal. We documented the DMARDs that were received by patients other than MTX, including hydroxychloroquine and immune-modulating biologic agents. Descriptive results were expressed as the median value and the distribution or the number (percentage) of patients according to the category of the variable (quantitative or qualitative).

## Results

**Fig 1:**
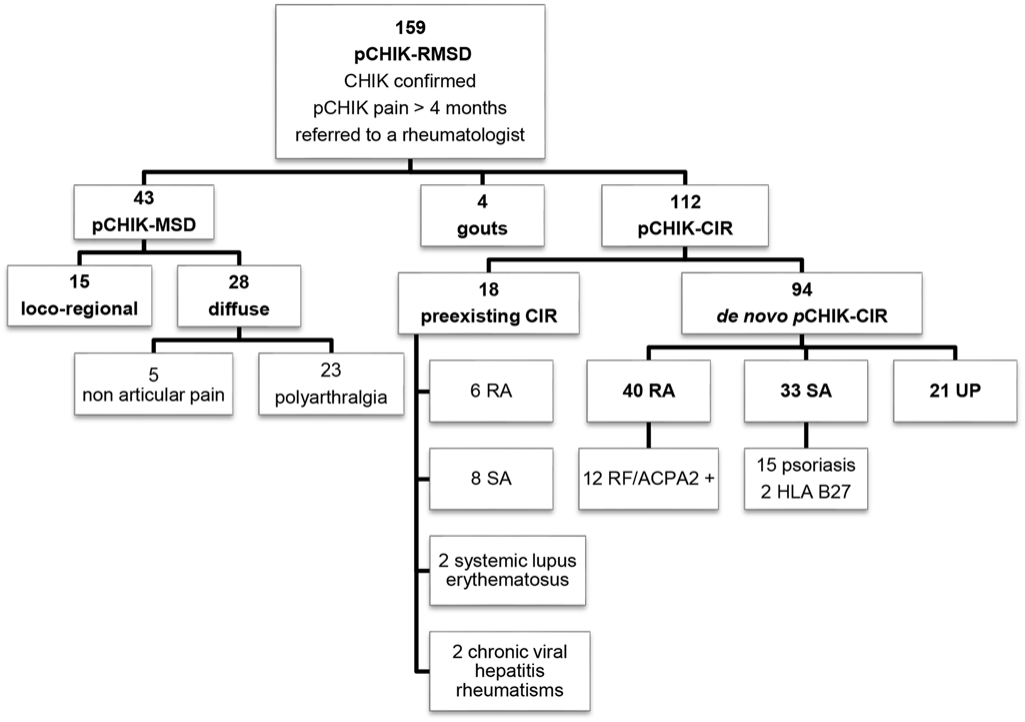
Nosologic flow-chart of patients referred to a rheumatologist for post-chikungunya (pCHIK) persistent rheumatic musculoskeletal pain, Saint-Denis, Reunion Island, 2006–2012.

**Fig 2:**
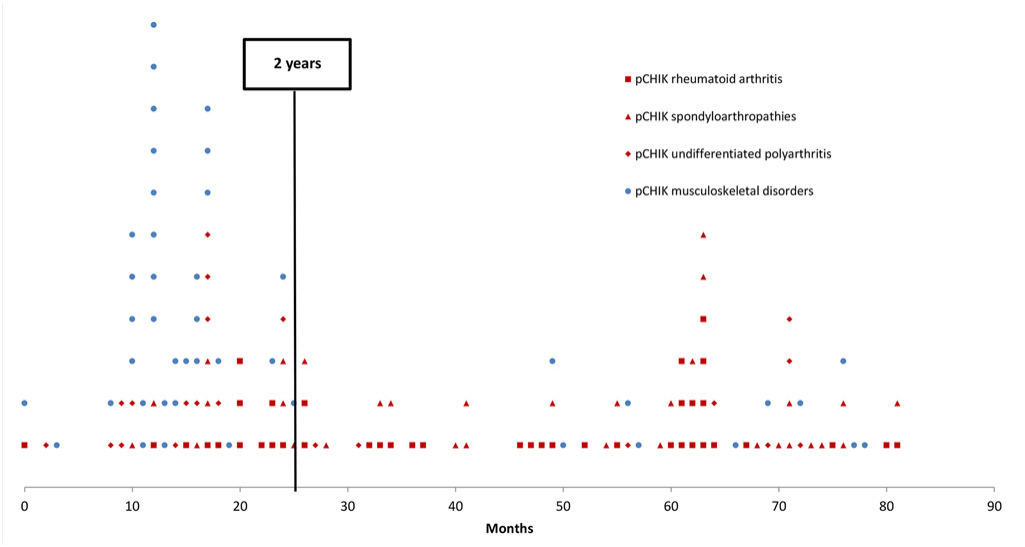
Time elapsed between chikungunya (CHIK) infection and the first visit to a rheumatologist for rheumatic or musculoskeletal disorders, Saint-Denis, Reunion Island, 2006–2012: musculoskeletal disorders versus chronic inflammatory rheumatisms.

A total of 159 patients with persistent pCHIK-RMSD were included in this study. The population was predominantly female (75%) and the median age was 51 years-old, ranging from 16 to 80 years-old. Repartition of pCHIK-RMSD is detailed in Fig. 1. Demographic characteristics and comorbidities are presented in Table 2, according to the patients’ CHIK-RMSD categories. There was no significant difference between these groups. In the entire cohort, 66% of patients reported a prolonged acute CHIK infection (fever> 10 days or symptoms> 3 weeks). The median time elapsed between CHIK infection and the first consultation with a rheumatologist was 2 years (median delay: 15.0 months for the MSD-group and 38.5 months for the CIR group) (Fig. 2); 80% of patients with pCHIK-MSD were referred within the first two years, whereas patients with pCHIK-CIR were regularly referred throughout the 6-year period, independent of the type of CIR.

**Table 2:** Characteristics of patients referred to a rheumatologist for rheumatic pains persisting after a confirmed chikungunya infection, Saint Denis, Reunion Island, 2006–2012.

Variables	STUDY COHORT	pCHIK-MSD		GOUT	PRE-EXISTING CIR	*de novo* pCHIK-CIR		
		Diffuse	Loco- regional			RA	SA	UP
**Number of patients**	159	28	15	4	18	40	33	21
Median age* in years [range]	51 [16–80]	52 [16–67]	56 [30–68]	46 [29–70]	46 [27–73]	49 [32–70]	49 [16–74]	59 [46–80]
**Sex ratio M/F**	38/121	6/22	4/11	3/1	4/14	10/30	10/23	1/20
**Tobacco use**	18 *(11%)*	2 (*5%*)	2 (*13%*)	1 (*25%*)	1 (*6%)*	5 (*13%*)	6 (*18%*)	1 (*5%)*
**Hypertension**	50 *(31%)*	8 (*19%*)	5 (*33%*)	2 (*50%*)	7 (*39%*)	11(*28%*)	8 (*24%*)	9 (*43%*)
**Diabetes mellitus**	17 *(11%)*	4 (*9%*)	1 (*7*%)	-	3 (*16*%)	4 (*10%*)	3 (*9%*)	2 (*10%*)
**Dysthyroidism**	8 *(5%)*	-	1 (*7*%)	-	2 (*11%*)	1 (*3*%)	1 (*3%*)	3 (*14%*)
**History of previous RMSD**	50 *(31%)*	10 (*23%*)	6 (*40*%)	1 (*25%*)	6 (*33%*)	8 (*20*%)	6 (*18%*)	13 (*62%*)
**Prolonged acute CHIK** **	105 *(66%)*	20 (*47%*)	6 (*40%*)	2 (*50%*)	10 *(56%)*	27 (*68%*)	25 (*76%*)	15 (*72%*)
Median time from acute CHIK to the first visit to rheumatologist (in months)	24 [0–81]	15 [0–78]	15 [8–56]	34 [13–81]	13 [7–77]	46 [0–81]	49 [10–81]	17 [2–71]

* Age in 2006 (at the acute stage);

** prolonged acute CHIK when fever >10 days or symptoms > 3 weeks.

CHIK: chikungunya;

pCHIK: post-CHIK;

CIR: chronic inflammatory rheumatism;

MSD: musculoskeletal disorders;

RA: rheumatoid arthritis;

RMSD: rheumatic musculoskeletal disorders;

SA: spondyloathropathy,

UP: undifferentiated polyarthritis.

All patients received painkillers as a first-line treatment, and 20% received vitamin D supplementation. The treatment history and medical features according to the different pCHIK-RMSD are summarized in Tables 3 and 4.

**Table 3:** Treatment history of patients referred to a rheumatologist for rheumatic or musculoskeletal pains persisting after a confirmed chikungunya infection, Saint Denis, Reunion Island, 2006–2012.

Variable	Total N *(%)*	pCHIK-MSD		GOUT	PRE-EXISTING CIR	*de novo* pCHIK-CIR		
		Diffuse	Loco-regional			RA	SA	UP
**Number of patients**	159	28	15	4	18	40	33	21
**Antalgics only**	7 *(4%)*	6 (*21%)*	1 *(7%)*	-	-	-	-	-
**Non steroid anti-inflammatory drugs**	61 (*38%)*	10 (*36%)*	7 *(47%)*	2 (*50%)*	9 (*50%)*	1 (*3%)*	20 (*61%)*	12 (*57%)*
**Oral corticosteroids exposure**	100 (*63%)*	20 (*71%)*	6 *(40%)*	1 (*25%)*	13 (*72*%)	32 (*80%)*	16 (*49%)*	12 (*57%)*
Withdrawal of corticosteroids *	*62*	*16*	*4*	*1*	*6*	*14*	*12*	*10*
**Hydroxychloroquine**	9 (*6*%)	5 (*18%)*	-	-	4 (*22%)*	-	-	-
**Methotrexate (MTX)**	80 (*50%)*	-	-	-	8 (*44%)*	40 (*100%)*	26 (*79%)*	6 (*29%)*
**Other DMARDs**	39 (*25%)*	-	-	-	13 (*72%)*	16 (*40%)*	10 (*30%)*	-
***Immune modulating biologic agents***	22 (*1%)*	-	-	-	10 (*55%)*	9 (*23%)*	3 *(9%)*	-
**Joint injections**	44 (*28%)*	8 (*29%)*	5 (*33%)*	3 *(75%)*	6 (*33%)*	10 (*25%)*	5 (*15%)*	7 (*33%)*
**Vitamin D**	34 (*21%)*	7 (*25%)*	3 (20%)	3 *(75%)*	5 (*28%)*	8 (*20%)*	6 (*18%)*	2 (*10%)*
**Urate-lowering therapy**	6 (4%)	-	1 (*7%)*	4 (*100%)*	1 (*6*%)	-	-	-
**Physiotherapy**	59 (*37%)*	10 (*36%)*	7 (*47%)*	3 (*75%)*	12 (*67%)*	11 (*28%)*	11 (*39%)*	5 *(24%)*

* No relapse 3 months after the termination of corticotherapy.

pCHIK: post-chikungunya;

MSD: musculoskeletal disorders;

CIR: chronic inflammatory rheumatism;

RA: rheumatoid arthritis;

SA: spondylarthropathy;

UP: undifferentiated polyarthritis;

DMARDs: disease-modifying antirheumatic drugs.

**Table 4:** Medical features in the different groups of post-chikungunya rheumatic and musculoskeletal disorders in patients referred to a rheumatologist for persisting pains, Saint-Denis, Reunion Island, 2006–2012.

	Total N *(%)*	pCHIK-MSD		GOUT	PRE-EXISTING CIR	*de novo* pCHIK-CIR		
		Diffuse	Loco-regional			RA	SA	UP
Number of patients	159	28	15	4	18	40	33	21
Tunnel syndromes	28 *(17%)*	7 *(16%)*	2 (*13%)*	2 (*50%)*	1 *(6%)*	8 (*20%)*	4 (*12*%)	4 (*19*%)
Limbs neurovascular disorders*	38 *(24%)*	9 (*21%*)	6 (*40%*)	2 *(50%)*	10 (56%)	5 (*13%*)	4 (*12*%)	2 (*10*%)
Radiological damage	58 *(37%)*	-	-	1 *(25%)*	9 (*50%)*	33 (*83%*)	13 (*39*%)	2 (*10*%)
Need for orthopedic brace	46 *(29%)*	5 (*18*%)	5 *(33%)*	1 *(25%)*	11 (*61%*)	15 (*38%*)	8 (*24*%)	1 (*5*%)
Job invalidity or adjustment	38 *(24%)*	1 *(4%)*	1 (*7%*)	1 (*25%*)	10 (*56%)*	11 (*28%*)	12 (*36*%)	2 (*10*%)
Reduction in daily activities	108 *(68%)*	13 *(46%)*	7 (*47*%)	2 (*50%*)	14 (*77*%)	34 (*85%*)	27 (*82*%)	11 (*52*%)
Psychological support	26 (*16%*)	5 (*18%*)	-	-	3 (*17*%)	7 (*18%*)	8 (*24*%)	3 (*15%)*

* Neurovascular disorders include Raynaud’s syndrome, acrosyndrome, algodystrophia, allodynia.

pCHIK: post-chikungunya;

CIR: chronic inflammatory rheumatism,

MSD: musculoskeletal disorders,

RA: rheumatoid arthritis,

SA: spondylarthropathy,

UP: undifferentiated polyarthritis.

The characteristics and specific management of the 43 cases of pCHIK-MSD are shown in Table 5. Only seven patients (15%) responded to the optimal painkiller treatment (using a second- or third-step analgesic if necessary, with the additional availability of a painkiller in the case of a painful episode). Among the 28 diffuse pCHIK-MSD, 22 consisted of distal polyarthralgia involving the hands (18 out of 22) and/or the feet (17 out of 22), typically associated with bilateral oedema of the extremities (about 50% of cases). This feature was frequently complicated by carpal or tarsal tunnel syndromes and paraesthesia that responded to a short course of corticotherapy (oral and/or injected into the joint) and sometimes improved with neuropathic pain medication, when indicated. Five patients with diffuse pCHIK-MSD (18%) received hydroxychloroquine without a self-reported benefit, while 20 out of 28 (70%) were efficiently treated with oral corticosteroids (16 out of 20 had a successful withdrawal of systemic corticotherapy without clinical relapse). Loco-regional pCHIK-MSD primarily involved proximal joints. In 11 cases, pCHIK-MSD presented as exacerbated pain in previously involved joints: arthrosis (n = 8) and injured areas (n = 3). Thirteen shoulder capsulitis or tendinitis required physiotherapy to limit stiffness and amyotrophy. Loco-regional CHIK-MSD mainly benefited from joint injections, physiotherapy and/or NSAIDs. Two of the 9 patients presenting with carpal tunnel syndromes developed algodystrophy (complex regional pain syndrome) after early surgery. Among the patients with inflammatory arthritis, 4 were diagnosed as early-onset crystalline polyarthritis, and they responded to gout treatment.

**Table 5:** Clinical characteristics and specific treatments of patients consulting for musculoskeletal pain (multiple joint inflammation excluded) persisting after a confirmed chikungunya infection, Saint-Denis, Reunion Island, 2006–2012.

	TYPE OF MSD	TOPOGRAPHY	ASSOCIATED MANIFESTATIONS	SPECIFIC TREATMENT associated with systematic painkillers
**DIFFUSE**	Polyarthralgia (n = 23)	Typically bilateral distal (22) involving hands (18) and/or feet (17): wrists, ankles, heels, MCP, PIP, MTP, exacerbated by use or standing	Distal edemas (10); Carpal syndrome & paresthesia (6); Dupuytren’s disease (1); Periostitis (painful bone pressure) (4); Shoulder capsulitis or tendinitis (7)	Oral/topic NSAIDs ± short course of oral corticotherapy or injection of corticoids in refractory join and if neuropathic pain association with tricyclic antidepressant, antiepileptic drugs or tramadol
		Spine (4)	Lombosciatalgia (3)	Discharge, splint, lombar belt
		Rhizomelic (shoulders) (3)	Amyotrophy (tighs) Myalgia	Physiotherapy, balneotherapy, thermal cure
		Knees (7)		
	Non articular pain (n = 5)	Muscles	Anxiety-depressive disorders (2); Tunnel syndrome (1); Tendonitis (1); Amyotrophy (1)	Pain killers, muscle relaxants, psychological follow-up
**LOCO-REGIONAL**	Exacerbation of arthrosis (n = 8)	Knee (4); Shoulder (2); Spine (2)	Amyotrophy (1); Sciatalgia (1); Nevralgia (C2); Tibial periostitis (1)	Oral NSAIDs and intra-articular corticosteroids during the acute pain phase when osteoarthrosis may be complicated by synovitis; Chondroprotectors intra-articularly (hyaluronic acid) and orally; Physiotherapy
	Mono arthritis (n = 2)	Shoulder (hydroxyapatite) (1); Hip (1)		NSAIDs; Physiotherapy; ultra-sounds, local injection
	Capsulitis (n = 5)	Shoulder	Stiffness (5)	Local injections; NSAIDs; Physiotherapy
	Tendinopathy (n = 11)	Rotator cuff (8); De Quervain's tendinosis (2); Tibialis anterior muscle (1)	Tendon rupture (1)	Injection of corticosteroids into the tendon Oral/topic NSAIDs; Splint to rest the thumb and wrist
	Periostitis (n = 7)	Ankle (4); Tibia (2); Wrist (1)		Oral/topic NSAIDs ± short course of oral corticosteroids
	Bursitis (n = 2)	Elbow (1); Shoulder (1)		NSAIDs, rest
	Osteonecrosis (n = 1)	Carpal bilateral	Algodystrophy	Splint; Physiotherapy
	Tunnel syndromes (n = 9)	Carpal (8); Ulnar (1)	Algodystrophy after surgery (2)	Splint; Oral/local corticosteroids
	Previously injured areas (exacerbation) (n = 3)	Sportive traumatism (2); Rachis fracture (1)	Foot stress fracture (1)	Physiotherapy; Balneotherapy; Biphosphonate if osteoporosis

*One patient may have several MSD*.

MCP: metacarpophalangeal;

PIP: proximal interphalangeal;

MTP: metatarsophalangeal;

NSAIDs: non steroidal anti-inflammatory drugs.

One hundred twelve patients met the criteria for CIR. Eighteen patients had pre-existing CIR (diagnosed or suspected) that was exacerbated immediately after CHIK infection, and the other 94 patients were considered pCHIK-CIR. Twenty-seven percent of patients with pCHIK-CIR reported the inability to work, and 77% reported a significant reduction in their daily activities. Half of the patients with pCHIK-CIR presented radiographical osteoarticular destructions or misalignments, mostly occurring in pCHIK-rheumatoid arthritis (RA) (83% of the cases). The median time from acute CHIK to the diagnosis of radiographical damage was 45 months (range 3–76). Thirteen patients (40%) with pCHIK-spondyloarthroarthritis (SA) had radiographical chondrolysis, 4 of these with sacroiliitis and 3 with bone erosions. Six other patients with SA had non-destructive arthritis with periosteal appositions and reconstructions.

Systemic corticotherapy was deemed necessary in 70% (13 out of 18) of pre-existing CIR, but complete withdrawal was achieved in only half of the cases. MTX was started in 8 patients with pre-existing CIR (3 RA, 4 SA and one systemic lupus previously under hydroxychloroquine), and it failed in 6 cases (3 RA, 3 SA), thereby leading to a switch to biologic DMARDs. A therapeutic escalation with immune-modulating biologic agents was required for 3 erosive RA previously controlled with MTX, and this appeared to have a beneficial effect; one patient with RA treated with infliximab was switched to etanercept, but remission occurred only after appropriate urate-lowering therapy, as the final diagnosis for their worsening condition was gout. For the 2 patients with untreated chronic viral hepatitis (B and C, one case each), hydroxychloroquine was ineffective in relieving joint inflammation, and consequently, sequential short courses of oral corticotherapy were given. Lastly, among the 4 patients who started hydroxychloroquine, 3 had to complete their treatment with immune-modulating biologic agents.

RA was the most common CIR in this cohort (16% pre-existing RA and 36% *de novo* pCHIK-RA) and was associated with the highest prevalence of osteoarticular damage, requirement for braces, and functional consequences (Table 4).

Among the 41 SA, we noticed a difference in clinical features: the 8 pre-existing SA presented as mostly ankylosing spondylitis with peripheral involvement (arthritis and/or enthesitis) triggered by CHIK, while the 33 *de novo* pCHIK-SA consisted of 15 cases of psoriatic polyarthritis (including 6 first occurrences of psoriasis) and 16 polyenthesitis (only two cases were HLA B27^+^). About one-third of patients with *de novo* pCHIK-CIR (14 RA, 11 SA and 3 UP) presented tenosynovitis of the wrist, hands and/or Achilles tendon, resulting in tunnel syndromes in 16 patients (17%).

Seventy-two pCHIK-CIR (77% with 100% of RA, 78% of SA and 30% of UP) received MTX at a mean weekly dose of 15 mg. Thirty six patients (50%) already had varying degrees of radiographical damage at the start of MTX treatment. With a median follow-up time of 21 months (a mean of 25 months), MTX led to a positive clinical response in 54 out of 72 patients (75% with 67% in RA, 80% in SA, 100% in UP); 7 cases developed bone destruction while undergoing MTX treatment, whereas recovery was achieved for 6 patients (3 RA and 3 SA). Nine patients had to promptly stop taking MTX due to side effects, which included 4 significant increases in transaminases, 2 digestive intolerances, one hair loss, one depression, one rash and cough); only 3 patients fully stopped MTX treatment due to a complete inefficacy. A total of 26 out of 94 patients received other DMARDs as a consequence of MTX contraindication or failure; 12 of these (9 RA, 3 SA) required immune-modulating biologic agents. Finally, algodystrophy after early carpal tunnel surgery was also reported in 3 more cases.

## Discussion

Alphaviral arthritides, including CHIK, have been blamed for causing protracted illnesses [3]. In light of the spreading of this disease, CHIK is increasingly becoming a burden on public health in endemic/epidemic areas worldwide. Considering both the incidence of CHIK disease over the last 10 years and the prevalence of symptoms persisting at least one year after the acute infection—from 4% in India and up to 66% in Italy [14,18]—the cumulative number of CHIK-infected individuals suffering from long-lasting pain and disabilities is estimated to be at least 1–2 million people. Recent studies of the long-term clinical outcome of pCHIK mostly addressed the prevalence of pCHIK-associated pain and incapacitation [12–14,18,20,21,34], but only a few detailed the clinical expression [15,16,23] and the results of treatment [25,27,30,31,40]. To date, physicians are still facing difficulties with the nosological approach to chronic patients, as they continue to seek the most efficient and non-deleterious treatments [41,42].

Our 6-year retrospective study confirmed that pCHIK-RMSD may last for years in the absence of specific and individualized approaches to treatment. It revealed a very late rheumatologic intervention, particularly in destructive pCHIK-CIR, that, in light of the positive MTX results we reported for pCHIK-CIR [43], should absolutely be shortened in analogy with RA. It is likely that the monocentric recruitment by rheumatologists overestimated the prevalence of pCHIK-CIR (about 5% of the patients at the chronic stage [13]). Nevertheless, this group has the worst functional prognosis and must be recognized early and managed specifically. Post-CHIK-CIR mostly accounted for pCHIK-RA and SA, which are well-defined, whereas pCHIK-undifferentiated polyarthritis (UP) is an emerging entity that can present a challenge to physicians. The characteristics of *de novo* pCHIK-RA that are clinically close to classical RA have been described after CHIK outbreaks in Reunion Island and India [15,23,30,31]. The prevalence of ACPA/RF positivity in our patients was 30%, and this is similar to that reported by others [30,31,44], with the exception of that of *Manimunda et al*., which reported a positivity rate of 5%, but after only a 10 month follow-up [15]. The rate in our study is, however, less than the 50% positivity that has been observed in classical RA populations [31,45]. Joint damage was very common in our series (about 80% of pCHIK-RA, 3 to 4 years after CHIK onset), higher than in pCHIK-SA (40%) and, of course, much higher than in pCHIK-UP (10%) as the logical consequence of the study’s definitions. These data on the destructiveness of pCHIK-RA are in accordance with those of *Bouquillard et al*. [31]. Like *Chopra et al*. [14] and *Mathew et al*. [16], we observed a high prevalence of psoriatic or pseudo-psoriatic (*i*.*e*. without psoriasis) polyarthritis. Interestingly, CHIK has also been suspected to trigger psoriasis [46]. An active search for psoriasis lesions on the skin, scalp and nails is thus recommended for all patients with suspected pCHIK-CIR. Radiographical signs in pCHIK-SA were chondrolysis with frequent periosteal appositions and reconstructions, but we also identified destructive erosive outcomes, as reported by *Malvy et al* [47]. Other forms of pCHIK-SA and enthesopathies were also identified [14], with a very low rate of HLA B27-positivity [47]. Lastly, pCHIK-UP is an entity with neither clinical or radiographical specificity, and is defined by excluding other rheumatisms (the alternative diagnosis could be a microcrystalline polyarthritis). PCHIK-UP requires a strict and prolonged follow-up in order to facilitate early detection of a switch to RA [48].

Considering the aforementioned data, any polyarticular inflammatory feature persisting more than 3 months after CHIK infection must suggest the potential for a diagnosis of pCHIK-CIR (see criteria in Table 1). Morning stiffness probably has a lower diagnostic value due to its high prevalence in the post-CHIK period, whereas synovitis and tenosynovitis are highly indicative of CIR (and therefore underscore the importance of ultrasonography to assess its severity and distinguish it from soft tissue oedema). Comparative and repeated radiographs of the hands and feet are necessary to detect damage.

This investigation sheds new light on the clinical polymorphism of pCHIK-RMSD, and it confirms the involvement of any part of the musculoskeletal system. These include synovium and cartilage, as well as bone, tendon and enthesis, which is consistent with the high CHIK virus (CHIKV) tropism for fibroblasts (including in tendinous insertions) and muscle satellite cells, as has been documented by others [49]. As previously reported [18,34], we also found a predominant incidence in females older than 45 years of age. Like *Mathew et al*. [16] and *Schilte et al*. [21], we found that chronic distal polyarthralgia, with or without swelling, was the most common MSD, with frequent knee involvement and high frequencies of tunnel syndromes and fatigue [20]. This clinical entity differs from remitting seronegative symmetric synovitis with pitting oedema (RS3PE) due to the lack of synovitis [50]. As discussed above, joint stiffness, even lasting more than 30 minutes, is often associated with delayed CHIK resolution [12,15,24]. In patients with pCHIK, diffuse non-articular pain, rhabdomyolysis caused by statins, depression or fibromyalgia should be investigated [16].

PCHIK-MSD treatment should be started with optimised painkillers in conjunction with physiotherapy and local infiltrations. NSAIDs, or short-course corticosteroids, may be added, as has been validated in long-lasting rheumatic disorders following Ross River virus infection [51,52]. Asthenia, psychological and daily life burdens must also be taken into account to avoid perpetuation of symptoms and to help with disease acceptance and recovery [15,16].

Neuropathic pain must be also identified and specifically treated with, for example, tricyclic antidepressants, antiepileptic drugs and/or tramadol. As indicated by responses to the “Douleur Neuropathique 4” (DN4) questionnaire, Ciampi de Andrade *et al*. found that 18% of patients complained of chronic pain 17 months after their acute CHIK infections [53].

Considering the high frequency of algodystrophy after early carpal tunnel syndrome surgery, we strongly advocate against surgery before the complete resolution of local inflammation, and we suggest alternative local treatment, such as cortisone injection.

**Fig 3:**
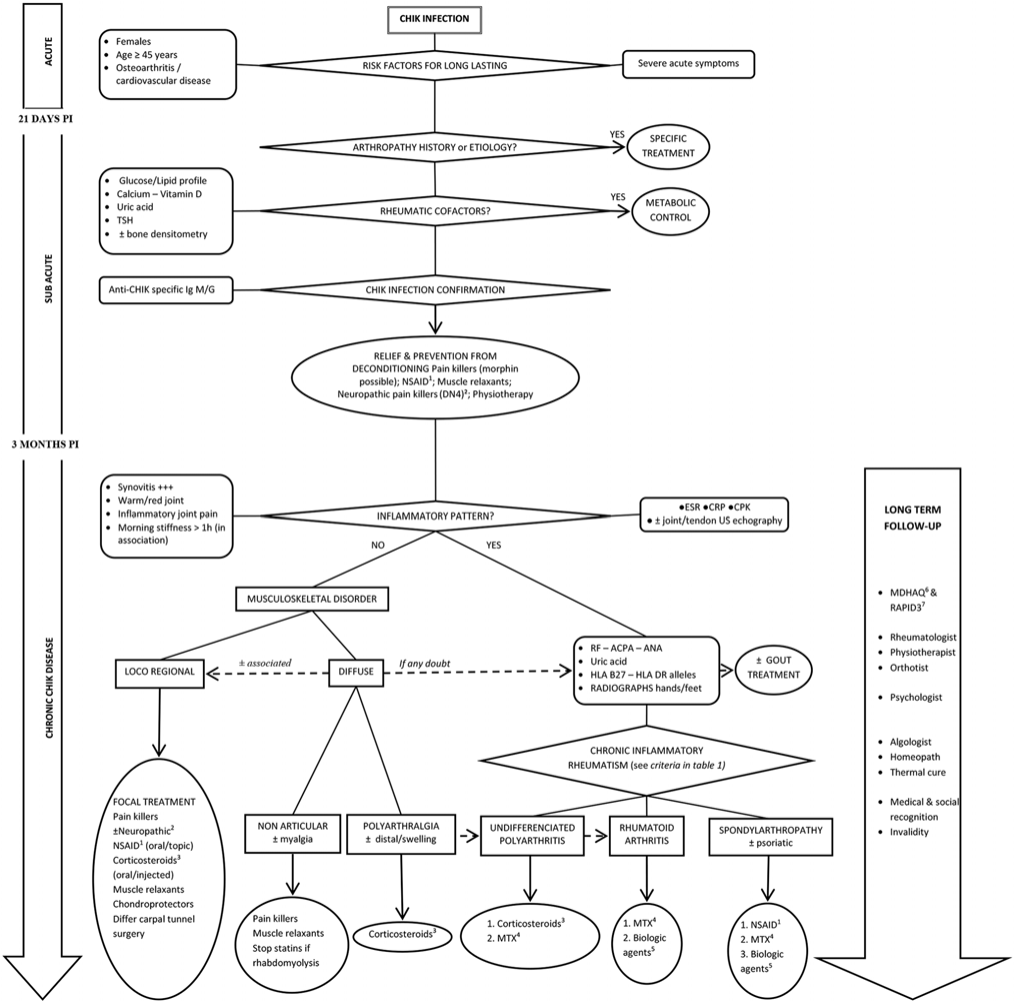
Proposal for a diagnostic and therapeutic algorithm to manage rheumatic and musculoskeletal disorders persisting after acute chikungunya (CHIK) infection, with the following abbreviations: ACPA = anti-citrullinated protein antibody; CHIK = chikungunya; CIR = chronic inflammatory rheumatisms; CPK = creatine phosphokinase; CRP = C-reactive protein; ESR = erythrocyte sedimentation rate; Ig = immunoglobulin; Pi = post-infection; MSD = musculoskeletal disorders; RF = rheumatoid factors; RMSD = rheumatic musculoskeletal disorders; TSH = Thyroid Stimulating Hormone; ^1^NSAID = non-steroidal anti-inflammatory drugs; ^2^DN4 = “Douleur Neuropathique 4” questionnaire. Neuropathic pain if ≥4/10 (sensitivity = 83% and specificity = 90%): use of tricyclic antidepressants, anti-epileptic drugs. ^3^Corticosteroids = [5–40] mg/day, short course (decrease and withdrawal within 6 months), associated with calcium and vitamin supplementation. ^4^MTX = methotrexate [7.5–25] mg/week orally or injected (notably if > 15mg/week); in the absence of contraindication (hepatic, pulmonary); in association with vitamin B9 (folate as folic acid or folinic acid) 5 to 10 mg/week 48 hours after MTX is taken; with weekly monitoring of complete blood count and monthly monitoring of liver and renal functions [60]. ^5^Immune-modulating biologic agents = rheumatologist prescription among anti-TNF (etanercept 25mg twice a week, infliximab 3–5 mg/kg/ 6–8 weeks, adalimumab 40 mg/ 2 weeks, golimumab 50mg/month), abatacept (inhibition of T-lymphocyte activation, 500–1000 mg/ 4 weeks), rituximab (depletion of B-lymphocytes, 1000 mg repeated at 2 weeks) and tocilizumab (inhibition of interleukin-6 receptor, 8 mg/ kg/ 2 weeks). ^6^MDHAQ = Multi-Dimensional Health Assessment Questionnaire [54]. ^7^RAPID 3 is significantly correlated with disease activity score (such as DAS28), and easily calculated in 10 second [55,56].

In light of the numerous types of pCHIK-RMSD reported by Mathew *et al*. in a large cohort 15 months after an outbreak in India [16] and the results described here, it is clear that diagnosis and treatment should be individualized and that, aside from painkillers and NSAIDs, no specific treatment can be universally recommended for all patients at the chronic pCHIK stage. We therefore provide an algorithm to assist with patient management (Fig. 3). We propose use of the Multi-Dimensional Health Assessment Questionnaire (MDHAQ), to be completed by the patient in the waiting room [54]. This questionnaire permits determination of the Routine Assessment of Patient Index Data (RAPID 3) score on a scale of 0–30, which can be used to follow the post-CHIK disease activity and the efficacy of treatments [55,56].

Most importantly, an “all is CHIK” approach should be avoided. A post-epidemic period and certain psychological backgrounds of the patients may promote over-diagnosis of chronic pCHIK disorders. The first step to unequivocal diagnosis entails an in-depth search for underlying conditions, so as to avoid misdiagnosing another disease that may be enhanced by CHIK and which requires a specific treatment. Pre-existing signs of chronic arthropathy, other comorbidities predisposing to pCHIK-RMSD and classical causes of chronic rheumatisms, such as hormonal disorders, autoimmune diseases, or other chronic infections (including hepatitis B and C), must also be considered. Depending on the outcome of these assessments, CHIK involvement can then be viewed as likely and serologically confirmed. Thus pCHIK-disorders are diagnosed when there is suggestive clinical expression, such as that compatible with data in the literature, following the exclusion of alternative diagnoses and biological proof of infection.

In our study, 106 (2/3) patients were serologically confirmed at a late stage, so that they were only positive for anti-CHIKV IgG. This may limit the causality of CHIK, since it could result from a previous infection. However, all patients complained of chronic pain starting immediately after a typical acute CHIK presentation that occurred during the first-ever documented CHIK outbreak in Reunion Island. In order to achieve a better accountability in light of the high prevalence of pCHIK rheumatic disorders and global expansion of the virus, we recommend implementation of serology as early as the subacute stage of the disease (i.e. starting at the 3^rd^ week of the disease) to confirm a recent CHIKV infection by the presence of IgM, which is usually detectable until 60 days post-infection [57].

Underlying mechanisms of chronic pCHIK disorders are poorly understood. As far as pCHIK-MSD is concerned, we hypothesize that local inflammation in synovium and tendons due to CHIK infection can compromise the osteo-myo-articular balance of previously susceptible joints, and that both overuse of the inflamed areas and loss of muscle strength hasten the degenerative process and related pain and stiffness. Whether CHIK is due to a viral trigger of dysimmunity in susceptible individuals, or mimics RA by sharing multiple inflammatory processes, remains to be ascertained [58]. Another prospective line of investigation, based on the viral sanctuary hypothesis (i.e. CHIK virus persistence in synovial macrophages) could result in novel, possibly curative therapeutic strategies, such as RNA silencing technology [59].

### Conclusion

CHIK virus is currently generating an epidemic of chronic rheumatism worldwide. Using a 6-year insight, this rheumatology case series from Reunion Island broadly describes clinical and therapeutic approaches to pCHIK’s long-lasting rheumatic disorders. We highlight the destructive pCHIK-CIR that affects a minority of patients, and we emphasize the importance of reducing the time necessary for the initiation of disease management by a medical specialist. Studies aimed at validating the efficacy of early use of MTX to prevent joint damage and long-term corticotherapy should be conducted in the future. Physiotherapy should also be evaluated in CHIK-MSD. Official guidelines for clinicians are necessary.
